# Maladie de Still en Afrique subsaharienne à travers dix observations du service de médecine interne du Centre hospitalier universitaire de Libreville (Gabon)

**DOI:** 10.48327/mtsi.v5i1.2025.629

**Published:** 2025-01-06

**Authors:** Josaphat IBA BA, Annick MFOUMOU, Ingrid NSENG-NSENG ONDO, Arthur KANGANGA EKOMY, Léonie Esther LEDAGA LENTOMBO, Marielle IGALA, Ulrich Davy KOMBILA, Jean Bruno BOGUIKOUMA

**Affiliations:** 1 Service de médecine interne, CHU de Libreville, BP 2228, Libreville, Gabon; 2 Service de rhumatologie, CHU de Libreville, BP 2228, Libreville, Gabon

**Keywords:** Maladie de Still, Hyperferritinémie, Corticothérapie, Méthotrexate, Libreville, Gabon, Afrique subsaharienne, Still's disease, Hyperferritinemia, Corticotherapy, Methotrexate, Libreville, Gabon, Sub-Saharan Africa

## Abstract

**Introduction:**

La maladie de Still (MS) est une affection inflammatoire systémique rare, plus fréquente dans la population infantile. Dans la forme de l'adulte, elle peut être primitive ou la résurgence de forme infantile. Nous rapportons 10 observations de maladie de Still dans la population gabonaise dans le but de confirmer son existence dans ce pays, et d'en rechercher les particularités.

**Matériel et méthodes:**

Il s'agit d'une étude rétrospective, descriptive et analytique réalisée dans le service de médecine interne du CHU de Libreville du 01/12/2003 au 31/12/2021, et prenant pour support les dossiers de patients hospitalisés dans ce service et/ou suivis en ambulatoire. Les patients retenus répondaient aux critères de Yamaguchi et Fautrel. Les données épidémiologiques, socio-économiques, cliniques, biologiques, morphologiques, immunologiques, thérapeutiques, évolutives, et saisonnières ont été détaillées.

**Résultats:**

Dix patients (4 hommes et 6 femmes), majoritairement des étudiants de 22 ans d’âge moyen ont été inclus. Le délai du diagnostic de la MS était de 31,3 mois avec, au premier plan, la fièvre dans 100 % des cas, l'atteinte articulaire et cutanée dans 80 % des cas et ORL dans 70 % des cas. Un syndrome inflammatoire, une hyperleucocytose à prédominance neutrophile, des signes hépatiques, et une hyperferritinémie coexistaient avec un bilan immunologique toujours négatif. La corticothérapie (n=10) couplée au méthotrexate per os d'emblée (n=1), ou lors de corticorésistance (n=4) était de règle. Nous déplorons le décès d'un patient et un perdu de vue.

**Discussion et conclusion:**

Le mode de présentation de la MS est comparable à celui dans la population caucasienne sur le plan clinique et biologique. Toutefois, notre étude confirme le faible nombre d'atteintes hépatiques, ganglionnaires et cardiaques, qui nécessiterait d’être confortée par d'autres études sur une cohorte plus importante. Le coût élevé de cette affection qui constitue un diagnostic d'exclusion peut en partie expliquer les difficultés de diagnostic de la MS.

## Abstract (en)

**Introduction:**

Introduction. Still's Disease (SD) is a rare systemic inflammatory disorder that is most common in children. In the adult form, it can be primary or the resurgence of an infantile form. We report 10 observations of Still's disease in the Gabonese population, with the aim of confirming its existence in this country an studying its specific features.

**Material and methods:**

This was a retrospective, descriptive and analytical study carried out in the Internal Medicine Department of the CHU of Libreville from 1.12.2003 to 31.12.2021, using the records of patients hospitalized in this department and/or followed up on an outpatient basis. The selected patients fulfilled the Yamaguchi and Fautrel criteria. Epidemiologic, socioeconomic, clinical, biological, morphologic, immunologic, therapeutic, evolutionary and seasonal data were detailed.

**Results:**

Ten patients (4 men and 6 women), mostly students with a mean age of 22 years, were included. The time to diagnosis of SD was 31.3 months, with fever in 100% of cases, joint and skin involvement in 80%, and ENT involvement in 70%. An inflammatory syndrome, predominantly neutrophilic hyperleukocytosis, hepatic signs, and hyperferritinemia coexisted with an immunologic work-up that was always negative. Corticosteroid therapy (n=10) combined with per os methotrexate from the beginning (n=1) or in cases of corticoresistance (n=4) was the rule. One patient died and one was lost to follow-up.

**Discussion and conclusion:**

The clinical and biological presentation of SD is comparable to that of the Caucasian population. However, our study confirms in the low incidence of liver, lymph node and heart damage, which needs to be confirmed by other studies in a larger cohort. The high cost of this disease, which is a diagnosis of exclusion, may partly explain the difficulties in diagnosing SD.

## Introduction

La maladie de Still (MS) est un rhumatisme inflammatoire systémique rare, d’étiologie inconnue, à début infantile, sans caractère familial, pouvant survenir ou se réactiver à l’âge adulte. Elle a été décrite pour la première fois en 1987 par le pédiatre anglais Georges Frederick Still [17] devant l'existence de polyarthrites séronégatives, non érosives, ne répondant pas aux critères classiques de la polyarthrite rhumatoïde. En 1971, Bywaters, interniste anglais, émettait l'hypothèse d'une entité clinique similaire chez 14 jeunes adultes présentant un tableau clinique semblable à celui de la forme systémique des arthrites juvéniles [2]. Les nouvelles recommandations européennes en 2023, présentées au congrès de l’EULAR *(European League of Associations for Rheumatism)* [8], considèrent que la forme pédiatrique et la forme de l'adulte représentent une seule et même entité, qu'il convient de nommer « maladie de Still ». Le diagnostic de MS repose sur les critères de Yamaguchi de 1992 [19], révisés par Fautrel *et al.* en 2002 [9]. L'association fièvre hectique, arthralgies et/ou arthrites, rash cutané, et hyperferritinémie > 1 000 ng/l, est habituellement suggestive de la maladie, après l'exclusion préalable d'une étiologie infectieuse, tumorale ou inflammatoire. En Afrique subsaharienne, la MS (comme de façon générale les maladies autoimmunes) demeure peu connue des praticiens, ce qui peut expliquer les données parcellaires dans cette région. Nous rapportons 10 observations de MS dans la population gabonaise pour confirmer l'existence de cette maladie dans ce pays, et en rechercher les particularismes.

## Matériel et méthodes

Il s'agit d'une étude rétrospective, descriptive, analytique et monocentrique réalisée dans le service de médecine interne du CHU de Libreville sur une période de 18 ans, du 01/12/2003 au 31/12/2021. Elle a pris pour support les dossiers de patients hospitalisés dans le service et/ou suivis en ambulatoire. Tous les patients inclus devaient avoir d'une part, un diagnostic de MS répondant aux critères de Yamagushi *et al.* [19] et/ou de Fautrel *et al.* [9] (Tableau I), réalisé en cours d'hospitalisation ou en ambulatoire, et avoir d'autre part fait l'objet d'un suivi. Les dossiers incomplets et les patients ne répondant pas aux précédents critères, étaient exclus. Les variables de l’étude détaillaient les données épidémiologiques, socio-économiques (identité, âge, sexe, profession), cliniques (fièvre, atteinte cutanée, articulaire et ORL), biologiques (NFS, CRP, transaminases), morphologiques (en fonction de l'orientation), immunologiques (autoanticorps antinucléaires, facteurs rhumatoïdes), thérapeutiques (antalgiques, et/ou antiinflammatoires non stéroïdiens, corticothérapie, immunosuppresseurs), évolutives (suivi, perdu de vu, décès), et saisonnières (en précisant la saison du diagnostic : grande saison sèche, de mai à septembre, petite saison sèche, de décembre à janvier, grande saison des pluies, de février à avril, et petite saison des pluies, d'octobre à novembre). Toutes les données étaient répertoriées sur une fiche de recueil de données, saisies à l'aide du logiciel Epi Info. Les données quantitatives étaient décrites à l'aide de moyenne et de médiane, et les données qualitatives à l'aide d'effectifs et de pourcentages.

**Tableau I:** Critères de Yamagushi et de Fautrel de la maladie de Still

Critères de Yamagushi (1992)	Critères de Fautrel (2002)
**Critères majeurs**	
1. Fièvre ≥ 39 °C, depuis une semaine ou plus	1. Pics fébriles ≥ 39 °C
2. Arthralgies depuis 2 semaines ou plus	2. Arthralgies ou arthrites
3. Rash cutané typique	3. Érythème transitoire ou fugace
4. Leucocytose ≥10 000/mm^3^ avec polynucléaires neutrophiles ≥ 80 %	4. Pharyngite
	5. Polynucléaires neutrophiles ≥ 80 %
	6. Fraction glycosylée de la ferritine sérique ≤ 20 %
**Critères mineurs**	
1. Pharyngite ou mal de gorge	1. Rash typique
2. Lymphadénopathie ou splénomégalie	2. Leucocytose ≥ 10 000/mm^3^
3. Élévation des transaminases	
4. Absence de facteur rhumatoïde ou d'anticorps antinucléaires	
**Critères d'exclusion**	
1. Absence d'infection (notamment sepsis profond et infection à EBV)	Aucun
2. Absence de néoplasie (notamment de lymphome)	
3. Absence de maladie inflammatoire (notamment de périartérite noueuse)	
**Diagnostic compatible :** pour au moins 5 critères dont 2 critères majeurs et pas de critères d'exclusion	**Diagnostic compatible** : pour 4 critères majeurs ou 3 critères majeurs et 2 critères mineurs

## Résultats

**Figure 1:**
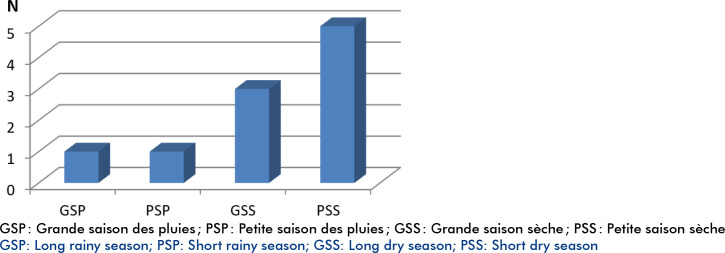
Période saisonnière de diagnostic

Dix patients (4 hommes et 6 femmes), de 26 ans d’âge médian (interquartiles IQ : 12-33; extrêmes : 2 et 45 ans) ont été inclus dans l’étude. L’âge médian chez les adolescents était de 11 ans (IQ : 10-12; extrêmes : 2 et 13 ans). Chez les adultes, l’âge médian était de 28 ans (IQ : 23-39; extrêmes : 19 et 45 ans). La tranche d’âge supérieur ou égal à 23 ans représentait 70 % de la population d’étude. La population estudiantine au sens large (élèves + étudiants) prédominait avec 6 cas, suivie des patients sans emploi (n=3), et de salariés (n=1). Une patiente signalait une tante avec polyarthrite rhumatoïde, et une seconde un asthme allergique dans une forme infantile. Huit cas ont été diagnostiqués en saison sèche (Fig. 1).

Sur le plan clinique, les données pertinentes se résumaient à une fièvre présente chez tous les patients, une atteinte articulaire et cutanée chez 8 d'entre eux, et une atteinte ORL chez 7 patients (Tableau II). Le délai diagnostic moyen de la MS était de 940 jours (extrêmes : 7 et 7 300 jours), avec un diagnostic inférieur ou égal à 14 jours chez 5 patients.

**Tableau II:** Données cliniques des patients

Mois et année de diagnostic	12-2003	09-2009	10-2009	01-2013	01-2018	12-2018	05-2019	12-2020	04-2021	09-2021	Synthèse
**Statut socio-économique**	Lycéen	Lycéenne	Salarié	Étudiante	Sans	Élève	Sans	Sans	Élève	Lycéen	
**Antécédents**	Sans	Asthme (enfance), atopie familiale	HTA	Sans	Sans	Sans	Angines à répétition (enfance)	Sans	Sans	Sans	Histoire immuno Sans 80 % Avec 20 %
**Âge**	23 ans	12 ans	45 ans	23 ans	33 ans	10 ans	39 ans	2 ans	13 ans	19 ans	
**Sexe**	H	F	F	F	F	H	F	H	F	H	4H+6F
**Fièvre**	40° Oui	40° Oui	39°5 Oui	39°7 Oui	Oui	39-40° Oui	39°7 Oui	Oui + convulsions	Oui (au long cours)	Oui	100 %
**Atteinte articulaire**	Oui	Oui	Oui	Oui diffuse	Oui	Oui	Oui diffuse			Oui	80 %
**Atteinte cutanée**	Oui	Urticaires	Érythème cuisses	Érythème fugace	Pseudo urticarienne	Non	Érythème fugace	Érythème fugace, lésions pseudo urticariennes	Non	Oui	80 %
**Atteinte ORL**	Non	Oui	Non	Oui	Oui	Oui	Oui	Oui	Non	Oui	70%
**Adénopathies**		Oui		Oui							
**Hépatomégalie**				Oui					Oui		20 %
**Atteinte séreuses**		Ascite				Pleurésie bilatérale			Ascite + pleurésie droite	Orchite	
**Atteinte cardiaque**	Non	Non	Non	Non	Non	Non	Non	Non	Non	Non	
**Délai du diagnostic**	730 jours	7 jours	14 jours	305 jours	7 jours	7 jours	7300 jours	2 jours	Non précisé	90 jours	Moyenne 940 jours /Average 940 days

H : Homme; F : Femme / M: Male; F: Female

Sur le plan biologique, une leucocytose existait chez 8 patients (taux moyen de 25 500/mm^3^, extrêmes : 7 500 et 60 000, médiane : 32 500/mm^3^) avec une prédominance de neutrophiles (9 cas), couplée à une anémie constante. Il existait un syndrome inflammatoire dans tous les cas où la CRP avait été réalisée (n=5), une perturbation du bilan hépatique chez 2 des 5 patients chez lesquels elle a été recherchée, et une hyperferritinémie dans 9 cas avec une moyenne de 23,3 N (extrêmes : normal et 9,6 N, médiane 3,7 N) (Tableau III). Les données morphologiques confirmaient une atteinte des séreuses dans 4 cas sur 7, chez les patients dont le bilan morphologique a été réalisé, une absence d'atteinte cardiaque et radiologique osseuse, et un tableau de polyadénopathies infra et centimétriques chez un patient (Tableau IV). Le bilan immunologique était négatif chez tous les patients, détaillé dans le Tableau V. Sur le plan thérapeutique, tous les patients avaient bénéficié d'une corticothérapie à base de prednisone (1 mg/ kg/jour), précédée dans 3 cas de bolus de méthyl prednisolone, couplé à du méthotrexate *per os* d'emblée chez un patient, ou en cas de corticorésistance (n=4) à la dose de 0,3 mg/kg/semaine soit 15 à 20 mg/semaine (Tableau VI), permettant une issue favorable chez 8 patients. Dans aucun cas, nous n'avons pu sevrer les patients en corticothérapie, avec une dose minimale efficace d'entretien journalière de 7,5 à 10 mg. Nous déplorons un décès en réanimation par syndrome d'activation lympho-histiocytaire (altération de l’état général fébrile, pancytopénie arégénérative, hyperferritinémie, augmentation de lactate déshydrogénase (LDH), et hémophagocytose au myélogramme), et un patient perdu de vue après 6 mois de suivi régulier malgré un contrôle satisfaisant sous traitement médical.

**Tableau III:** Données biologiques des patients

Âge	2 ans	10 ans	12 ans	13 ans	19 ans	23 ans	23 ans	33 ans	39 ans	45 ans	Synthèse
Rubrique																						
Leucocytes (/mm^3^)	15 460	47 950	5 700	60 000	17 900	15 000	38 000	25 200	20 800	9 120	25 547,7
Neutrophiles	71,50 %	75 %	61,30 %	40 %	85 %	72 %	75 %	72 %	80%	72%	70%
Hémoglobine	11,6	8,6	11,8	7,5	10,5	6,1	6,7	9,7	11,3	10,3	9,3
Plaquettes (/mm^3^)	361000	671000	344000	492000	408000	495000	252000	452000	193 000	275000	374 875
CRP (mg/l)		29,5 N			8,9 N	94 N		6,25 N	81,3 N		43,9 N
LDH		3,1 N						1,3 N		1,9 N	
ASAT			N	2,2 N				N	N	2 N	
ALAT			N	2,4 N				N	N	N	
CPK	1,1 N		N	N			N	N	N	1,3 N	N
Ferritinémie	Normal sous aspirine	29,9 N	1,6 N	1,7 N	2,6 N	40 N	99,6 N	1,4 N	4,8 N	6,5 N	23,3 N
Ferritine glycosylée		8 %									

N : Nombre de fois la normale; CRP : Protéine C réactive; CPK : Créatinine phosphokinase; LDH : Lacticodéshydrogénase; ALAT : Alanine-amino-transférase; ASAT : Aspartate-amino-transférase

**Tableau IV:** Données morphologiques des patients

Âge	2 ans	10 ans	12 ans	13 ans	19 ans	23 ans	23 ans	33 ans	39 ans	45 ans	Synthèse
Rubrique											
Rx Thorax	Non réalisé	Pleurésie bilatérale	N	Pleurésie droite	N	Pleurésie droite	Non réalisé	Non réalisé	N	N	Pleurésie 42,80%
Rx articulaires						N			N		N
Échocardiographie					N	N			N		N
Échographie abdominale			HMG	Ascite							HMG 20%
Tomodensitométrie			Poly-ADP								ADP 10%

N : Normale; HMG : Hépatomégalie; ADP : Adénopathie

**Tableau V:** Données immunologiques des patients

Âge	2 ans	10 ans	12 ans	13 ans	19 ans	23 ans	23 ans	33 ans	39 ans	45 ans
Rubrique										
Anticorps antinucléaires	N	N	N	N	N	N	N	N	N	N
Anti DNA natifs			N	N	N	N		N	N	
Anticorps nucléaires solubles			N	N		N		N	N	N
Anticorps antiphospholipides		N	N	N						
ANCA	N		ANCA	N			N		N	
Facteur rhumatoïde	N	N							N	
Anti-CCP			N						N	N

N : Négatif ANCA : Autoanticorps dirigés contre le cytoplasme des neutrophiles; Anti-CCP : Anticorps anti peptides cycliques citrulinés

**Tableau VI:** Données thérapeutiques des patients

Âge	2 ans	10 ans	12 ans	13 ans	19 ans	23 ans	23 ans	33 ans	39 ans	45 ans
Rubrique										
Bolus de MP IV (x 3 jours)	480 mg				40 mg		750 mg	120 mg		
Prednisone per os	0,7 mg/kg/j	1 mg/kg/j	1 mg/kg/j	1 mg/kg/j	1 mg/kg/j	1 mg/kg/j	1 mg/kg/j	1 mg/kg/j	1 mg/kg/j	1 mg/kg/j
Méthotrexate per os	15 mg/ semaine									
Aspirine Per os	Non	Non	Non	Non	Non	Non	Non	Non	Non	Non
AINS						Oui				
Récurrences	CR 15 mg	Non	Non	Non	Non	CR 15 mg	Oui+DCD	CR 15 mg	Non	CR 30 mg
Perdu de vue		Oui			Oui				Oui	

mg : Milligramme; kg : Kilogramme; DCD : Patient décédé; CR : Corticorésistance; MP iv : Méthyl prednisolone intraveineux; PO : per os; AINS : Anti inflammatoire non stéroïdien

## Discussion

La MS demeure certainement sous-estimée en Afrique subsaharienne devant le peu d'observations retrouvées, contrastant avec une absence de prédisposition génétique clairement établie de la maladie [11]. En effet, les données disponibles sont surtout issues de populations caucasiennes ou asiatiques et il n'existe que très peu de données dans la population noire ou d'ascendance africaine. À cela, il faut rajouter le coût élevé du bilan concourant au diagnostic de la maladie dans un continent où le système d'assurance maladie n'est pas encore établi pour le plus grand nombre. Le Gabon a la chance de bénéficier d'une assurance maladie nationale depuis 2007 prenant 80 % du coût de prise en charge ambulatoire et hospitalière [14], ce qui nous a permis d'aboutir au diagnostic de maladie de Still. Dans l'itinéraire diagnostic des patients, nous n'avons pas retrouvé de consultation chez des tradithérapeutes, dont le traitement est à base de scarifications et de décoctions traditionnelles. La MS constitue un diagnostic d'exclusion, imposant avant d’être évoquée, d’écarter préalablement une étiologie infectieuse, tumorale et auto-immune.

La MS provoque une inflammation systémique, au stade précoce de la maladie ou lors de rechutes et de récurrences, résultant de l'activation et de la sécrétion de cytokines pro-inflammatoires, notamment d'interleukine (IL)-1, d’IL-18 et d’IL-6 [4, 10, 13]. Neuf de nos patients (un étant lors du diagnostic sous acide acétylsalicylique) avaient une hyperferritinémie comme classiquement retrouvée dans cette maladie. Ce taux très élevé de ferritine (mais également d’IL-8, IL-6, IL-18 et TNF-α) rend compte d'une activation marquée des macrophages lesquels, lorsqu'ils sont activés, synthétisent de l’IL-18 qui jouerait un rôle clé dans la pathogénie de la maladie [1].

De façon générale, pour les mêmes critères diagnostics [19], type d’étude (rétrospective), et type de population [5, 16], notre population demeure plus jeune que celle observée par d'autres auteurs au Cameroun, où l’âge moyen des patients est de 28 ans [16], au Sénégal où il est de 43 ans [5] et en Tunisie où il est de 35,4 ans [3]. Deux patients sur 3 sont diagnostiqués avant 40 ans [15], alors que notre série en compte 9 sur 10. Dans 10 à 15 % des cas, la MS peut être la résurgence d'une maladie de Still de l'enfance. Nous n'avons pas eu dans l'histoire de nos patients, d'observation de MS survenue dans l'enfance, qui aurait pu faire évoquer cette résurgence de forme infantile. Nous retrouvons 8 observations sur 10 de MS en saison sèche, mais l'influence saisonnière sur l'apparition de cette maladie n'a jamais été étudiée en Afrique subsaharienne.

**Figure 2:**
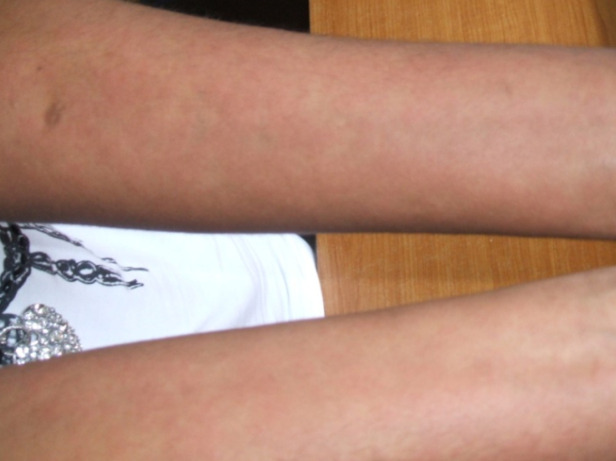
Lésions cutanées de maladie de Still sur peau noire

Actuellement, les sociétés savantes s'accordent pour retenir la MS de l'adulte et sa forme juvénile comme une seule et même maladie, avec une même présentation clinique [8]. On distingue les MS à tropisme articulaire et les MS avec atteinte systémique. Sur le plan clinique, la MS se caractérise par une triade associant : a) fièvre élevée et hectique, b) éruption cutanée évanescente, et c) atteinte articulaire à type d'arthralgies ou d'arthrites. La fièvre d'allure septique avec frissons demeure une constante dans les études d’Afrique subsaharienne [5, 16], de même que l'atteinte articulaire (8 cas dans notre série), cutanée (8 cas) et ORL, retrouvée chez 7 de nos patients. Dans sa forme typique, l'atteinte cutanée au cours de la maladie de Still associe sur peau caucasienne de petites macules ou maculo-papules, de couleur rose saumonée, non prurigineuses, fugaces, siégeant à la racine des membres et apparaissant au moment des poussées fébriles. Cette atteinte cutanée est plus difficile à mettre en évidence sur peau noire, et peut prendre à défaut mêmes des spécialistes aguerris (Fig. 2). Ceci pourrait expliquer le faible nombre d'atteintes cutanées retrouvées dans la série de Singwe-Ngandeu *et al.* de 16,6 % [16].

Nous notons dans notre série d'une part une plus faible prévalence des atteintes hépatiques et ganglionnaires (rattachées à la MS devant leur régression sous corticothérapie), et, d'autre part, une absence d'atteinte cardiaque. Ces atteintes sont pourtant fréquentes au cours de la MS.

Biologiquement, aucune perturbation n'est spécifique de la MS, mais trois sont très évocatrices : 1) un syndrome inflammatoire, 2) une hyperleucocytose à polynucléaires neutrophiles et 3) une hyperferritinémie avec une fraction glycosylée inférieure à 20 %. À l’état normal, la forme glycosylée représente 60 à 80 % de la forme circulante. Au cours de la maladie de Still, le pourcentage de la fraction glycosylée est plus bas comparé aux autres pathologies inflammatoires et auto-inflammatoires [7]. Cette fraction apparaît diminuée en phase active mais aussi durant les phases de rémission. La combinaison hyperferritinémie 5 fois supérieure à la normale, couplée à une fraction glycosylée < 20% correspond à une spécificité de 93 % et une sensibilité de 40 % pour la maladie de Still [18]. Malheureusement, dans notre étude, du fait de son coût élevé par rapport aux possibilités financières des patients, ce marqueur biologique n'a pu être dosé que chez un seul patient. De ce fait, il nous semble que les critères de Yamaguchi *et al.* [19], demeurent plus accessibles pour le diagnostic de MS en Afrique subsaharienne que ceux de Fautrel *et al.* [9].

Sur le plan thérapeutique, les anti-inflammatoires non stéroïdiens (AINS), efficaces dans la MS chez l'enfant, ne semblent efficaces que chez 20 % des adultes atteints de MS dans les séries européennes [12]. C'est pourquoi notre choix s'est tourné d'emblée vers une corticothérapie isolée pouvant être associée au méthotrexate. La corticothérapie a été utilisée à la dose de 0,5 à 1 mg/ kg/jour de prednisone chez tous nos patients, avec introduction de méthotrexate à la dose de 0,3 mg/ kg/jour, dans un but d’épargne cortisonique, mais également dans les atteintes articulaires du fait de son efficacité démontrée, en cas de corticorésistance (4/10 patients). Les récurrences sont moins importantes lorsque le méthotrexate est d'emblée associé à la corticothérapie (8 % dans la série de Diallo *et al.*) [5]. En cas d’échec thérapeutique, la discussion se porte sur l'instauration de biothérapies. Ces thérapeutiques, quoique novatrices et très efficaces, trouvent leurs limites en Afrique subsaharienne du fait de leur coût élevé d'une part, et de la résurgence de tuberculose qu'elles engendrent dans un continent où cette affection sévit à l’état d'endémie, d'autre part [6].

Dans tous les cas, nos patients n'ont pas pu être sevrés des thérapeutiques (corticothérapie et méthotrexate). Quatre de nos patients ont présenté une corticorésistance imposant l'adjonction de méthotrexate, et nous déplorons un décès par syndrome d'activation lympho-histiocytaire.

## Conclusion

Les données de la MS en Afrique subsaharienne demeurent parcellaires. Notre préférence pour le diagnostic va vers les critères de Yamagushi *et al.* [19]. Notre étude confirme un faible nombre d'atteinte hépatique et ganglionnaire, ainsi qu'une absence d'atteinte cardiaque. Elle nécessite d’être confortée par d'autres études sur une cohorte plus importante. De même, la fréquence du diagnostic en saison sèche (saison de faibles températures) devrait être étudiée sur une plus grande cohorte.

## Source de financement

Ce travail n'a bénéficié d'aucune source de financement.

## Contribution des auteurs et autrices

Iba Ba Josaphat : conception et rédaction Mfoumou Annick Flore : rédaction et relecture Nseng Nseng Ondo Ingrid : relecture et collecte de données

Kanganga Ekomy Arthur : recherche bibliographique, relecture

Ledaga Lentombo Léonie Esther, Igala Marielle, et Kombila Ulrich Davy : relecture Boguikouma Jean Bruno : conception, relecture, approbation de la version finale

## Liens d'intérêts

Les auteurs et autrices déclarent ne pas avoir de liens d'intérêts.
