# Supplementary material for: Still's disease in sub-Saharan Africa through ten observations from the internal medicine department of Libreville University Hospital (Gabon)
Source: Med Trop Sante Int. 2025 Jan 6;5(1):mtsi.v5i1.2025.629. [Article in French] doi: 10.48327/mtsi.v5i1.2025.629 (PMC12001992; doi:10.48327/mtsi.v5i1.2025.629)
Supplement: Related texts

# Research article: Still's disease in sub-Saharan Africa through ten observations from the internal medicine department of Libreville University Hospital (Gabon)

## Introduction

Still's disease (SD) is a rare systemic inflammatory rheumatic disease of unknown etiology, with infantile onset and no familial features, which may manifest or reactivate in adulthood. It was first described in 1987 by the English pediatrician Georges Frederick Still [17], who observed a seronegative, non-erosive polyarthritis that did not meet the classic criteria for rheumatoid arthritis. In 1971, Bywaters, an English internist, hypothesized a similar clinical entity in 14 young adults with a clinical picture similar to the systemic form of juvenile arthritis [2]. New European recommendations in 2023, presented at the EULAR (European League of Associations for Rheumatism) Congress [8], consider the pediatric and adult forms to be a single entity called “Still's disease”. The diagnosis is based on the 1992 Yamaguchi criteria [19], revised by Fautrel *et al.* in 2002 [9]. The combination of hectic fever, arthralgia and/or arthritis, rash, and hyperferrit-inemia > 1,000 ng/L is usually suggestive of the disease, after exclusion of infectious, tumoral, or inflammatory etiologies. In sub-Saharan Africa, SD (and autoimmune diseases in general) remains poorly understood by practitioners, which may explain the paucity of data in this region. We report 10 observations of SD in the Gabonese population to confirm the existence of this disease in this country and to study its peculiarities.

## Material and methods

This is a retrospective, descriptive, analytical and monocentric study carried out in the Internal Medicine Department of the CHU of Libreville over a period of 18 years, from 12/01/2003 to 12/31/2021. It was based on the records of patients admitted to the department and/or followed up in the outpatient clinic. All patients included had to be diagnosed with SD according to the criteria of Yamagushi *et al.* [19] and/or Fautrel *et al.* [9] (Table I), either during hospitalization or as an outpatient, and had to be followed up. Incomplete records and patients who did not meet the above criteria were excluded. Study variables detailed epidemiologic, socioeconomic (identity, age, sex, occupation), clinical (fever, skin, joint and ENT involvement), biological (CBC, CRP, transaminases), morphologic (according to orientation), immunologic (antinuclear autoimmune immunologic (antinuclear autoantibodies, rheumatoid factors), therapeutic (analgesics and/or non-steroidal anti-inflammatory drugs, corticosteroid therapy, immunosuppressants), evolutionary (follow-up, loss of vision, death), and seasonal (specifying the season of diagnosis: Long dry season, May to September; Short dry season, December to January; Long rainy season, February to April; Short rainy season, October to November). All data were recorded on a data collection sheet and entered using Epi Info software. Quantitative data were described using mean and median, and qualitative data were described using numbers and percentages.

**Table I:** Yamagushi and Fautrel criteria for Still's disease

Yamagushi criteria (1992)	Fautrel criteria (2002)
**Major criteria**	
1. Fever ≥ 39°C, for a week or more	1. Fever peaks ≥ 39°C
2. Arthralgia for 2 weeks or more	2. Arthralgia or arthritis
3. Typical skin rash	3. Transient or fleeting erythema
4. Leukocytosis ≥10,000/mm^3^ with neutrophils ≥ 80%	4. Pharyngitis
	5. Neutrophils ≥ 80%
	6. Glycosylated serum ferritin fraction ≤ 20%
**Minor criteria**	
1. Pharyngitis or sore throat	1. Typical rash
2. Lymphadenopathy or splenomegaly	2. Leukocytosis ≥ 10,000/mm^3^
3. Elevated transaminases	
4. Absence of rheumatoid factor or antinuclear antibodies	
**Exclusion criteria**	
1. Absence of infection (including deep sepsis and EBV infection)	None
2. Absence of neoplasia (especially lymphoma)	
3. Absence of inflammatory disease (especially periarteritis nodosa)		
**Compatible diagnosis**: for at least 5 criteria including 2 major criteria and no exclusion criteria	**Compatible diagnosis**: for 4 major criteria or 3 major and 2 minor criteria

## Results

Ten patients (4 males and 6 females) with a median age of 26 years (IQ interquartile range: 12-33; extremes: 2 and 45 years) were included in the study. In adolescents, the median age was 11 years (IQ: 10-12; extremes: 2 and 13 years). In adults, the median age was 28 years (IQ: 23-39; extremes: 19 and 45 years). Those aged 23 years and over accounted for 70% of the study population. The student population in the broad sense (pupils + students) predominated with 6 cases, followed by unemployed patients (n=3) and employed patients (n=1). One patient reported an aunt with rheumatoid arthritis, and a second patient reported allergic asthma in an infantile form. Eight cases were diagnosed during the dry season (Fig. 1).

**Figure 1:** Seasonal diagnostic period

Clinically relevant data were fever in all patients, joint and skin involvement in 8 patients, and ENT involvement in 7 patients (Table II). The mean time to diagnosis of SD was 940 days (extremes: 7 and 7,300 days), with a diagnosis of 14 days or less in 5 patients.

**Table II:** Clinical data of patients

Month and year of diagnosis	12-2003	09-2009	10-2009	01-2013	01-2018	12-2018	05-2019	12-2020	04-2021	09-2021	Synthesis
**Socio-economic status**	High school student	High school female student	Employee	Female student	Without	Student	Without	Without	Student	High school student	
**Case history**	Without	Asthme (enfance), atopie familiale / Asthma (childhood), familial atopy	HTA	Without	Without	Without	Angines à répétition (enfance) / Recurrent tonsillitis (childhood)	Without	Without	Without	immuno history - Without 80% - With 20% /
**Age**	23 years	12 years	45 years	23 years	33 years	10 years	39 years	2 years	13 years	19 years	
**Gender**	H / M	F / F	F / F	F / F	F / F	H / M	F / F	H / M	F / F	H / M	4H+6F / 4M+6F
**Fever**	40° Yes	40° Yes	39°5 Yes	39°7 Yes	Yes	39-40° Yes	39°7 Yes	Yes + convulsions	Yes (long-term)	Yes	100 %
**Joint involvement**	Yes	Yes	Yes	Yes diffuse	Yes	Yes	Yes diffuse			Yes	80 %
**Skin involvement**	Yess	Urticaria	Thigh erythema	Fleeting erythema	Urticarialike lesions	No	Fleeting erythema	Fleeting erythema, urticarialike lesions	No	Yes	80 %
**ENT involvement**	No	Yes	No	Yes	Yes	Yes	Yess	Yes	No	Yes	70 %
**Adenopathy**		Yes		Yes							
**Hepatomegaly**				Yes					Yes		20 %
**Serosal involvement**		Ascites				Bilateral pleurisy			Ascites + right pleuresis	Orchitis	
**Cardiac involvement**	No	No	No	No	No	No	No	No	No	No	
**Time to diagnosis**	730 days	7 days	14 days	305 days	7 days	7 days	7300 days	2 days	Not specified	90 days	Average 940 days

H : Homme; F : Femme / M: Male; F: Female

Biologically, 8 patients had leukocytosis (mean 25,500/mm^3^, range 7,500 to 60,000, median 32,500/mm^3^), with neutrophils predominating (9 cases), associated with persistent anemia. An inflammatory syndrome was present in all cases in which CRP was performed (n=5), liver dysfunction in 2 of the 5 patients in whom it was performed, and hyperferritinemia in 9 cases with a mean of 23.3 N (extremes: normal and 9.6 N, median 3.7 N) (Table III). Morphologic data confirmed serosal involvement in 4 of 7 cases, absence of cardiac and bone radiologic involvement in patients who underwent morphologic examination, and infra- and centimetric polyadenopathy in one patient (Table IV). Immunologic tests were negative in all patients, as shown in Table V. Therapeutically, all patients received prednisone-based corticosteroids (1 mg/kg/day), preceded in 3 cases by a methylprednisolone bolus, coupled with immediate per os methotrexate in one patient or, in case of corticoresistance (n=4), at a dose of 0.3 mg/kg/week, i.e. 15 to 20 mg/week (Table VI), resulting in a favorable outcome in 8 patients. In no case were we able to wean patients off corticosteroids with a minimum effective daily maintenance dose of 7.5 to 10 mg. One patient died in the intensive care unit due to lymphohistiocytic activation syndrome (altered febrile general condition, areregenerative pancytopenia, hyperfer-ritinemia, elevated lactate dehydrogenase (LDH), and hemophagocytosis on myelogram), and one patient was lost to follow-up at 6 months despite satisfactory control with medical treatment.

**Table III:** Biological data of patients

Age	2 years	10 years	12 years	13 years	19 years	23 years	23 years	33 years	39 years	45 years	Synthesis
Items											
Leukocytes (/mm^3^)	15 460	47 950	5 700	60 000	17 900	15 000	38 000	25 200	20 800	9 120	25 547,7
Neutrophils	71,50 %	75 %	61,30 %	40 %	85 %	72 %	75 %	72 %	80%	72%	70%
Hemoglobin (g/dl)	11,6	8,6	11,8	7,5	10,5	6,1	6,7	9,7	11,3	10,3	9,3
platelet (/mm^3^)	361000	671000	344000	492000	408000	495000	252000	452000	193 000	275000	374 875
CRP (mg/l)		29,5 N			8,9 N	94 N		6,25 N	81,3 N		43,9 N
LDH		3,1 N						1,3 N		1,9 N	
ASAT			N	2,2 N				N	N	2 N	
ALAT			N	2,4 N				N	N	N	
CPK	1,1 N		N	N			N	N	N	1,3 N	N
Ferritinemia	Normal on aspirin	29,9 N	1,6 N	1,7 N	2,6 N	40 N	99,6 N	1,4 N	4,8 N	6,5 N	23,3 N
Glycosylated ferritin		8 %									

N: Number of times normal; CRP: C-reactive protein; CPK: Creatinine phosphokinase; LDH: Lactic dehydrogenase; ALAT: Alanine aminotransferase; ASAT: Aspartate aminotransferase

**Table IV:** Morphological data of patients

Age	2 years	10 years	12 years	13 years	19 years	23 years	23 years	33 years	39 years	45 years	Synthesis
Items											
Thorax X-ray	Not performed	Bilateral pleurisy	N	Right pleurisy	N	Right pleurisy	Not performed	Not performed	N	N	Pleuresis 42,80%
Joint X-ray						N			N		N
Echocardiography					N	N			N		N
Abdominal ultrasound			HMG	Ascites + HMG							HMG 20%
Computed tomography			Poly-ADP								ADP 10%

N : Normale; HMG : Hépatomégalie; ADP : Adénopathie

**Table V:** Immunological data of patients

Age	2 years	10 years	12 years	13 years	19 years	23 years	23 years	33 years	39 years	45 years
Items										
Antinuclear antibodies	N	N	N	N	N	N	N	N	N	N
Native anti DNA			N	N	N	N		N	N	
Soluble nuclear antibodies			N	N		N		N	N	N
Antiphospholipid antibodies		N	N	N						
ANCA	N		ANCA	N			N		N	
Rheumatoid factor	N	N							N	
Anti-CCP			N						N	N

N: Negative ANCA: Antineutrophil cytoplasmic antibodies; Anti-CCP: Anti-cyclic citrullinated peptide

**Table VI:** Therapeutic data of patients

Age	2 years	10 years	12 years	13 years	19 years	23 years	23 years	33 years	39 years	45 years
Items										
Bolus of MP IV (x 3 days)	480 mg				40 mg		750 mg	120 mg		
Per os prednisone	0,7 mg/kg/j	1 mg/kg/j	1 mg/kg/j	1 mg/kg/j	1 mg/kg/j	1 mg/kg/j	1 mg/kg/j	1 mg/kg/j	1 mg/kg/j	1 mg/kg/j
Per os mehotrexate	15 mg/week									
Per os Aspirin	No	No	No	No	No	No	No	No	No	No
NSAID						Oui				
Recurrences	CR 15 mg	No	No	No	No	CR 15 mg	Yes+DCD	CR 15 mg	No	CR 30 mg
Lost to follow-up		Yes			Yes				Yes	

mg: Milligram; kg: Kilogram; DCD: Patient deceased; CR: Corticoresistance; MP iv: Intravenous methyl prednisolone; PO: per os; NSAID: Non-steroidal anti-inflammatory drug

## Discussion

SD is certainly underestimated in sub-Saharan Africa, given the paucity of observations and the absence of a clearly established genetic predisposition to the disease [11]. In fact, the available data are mainly from Caucasian or Asian populations, and there are very few data from the black population or people of African descent. This is compounded by the high cost of the work-up required to diagnose the disease in a continent where health insurance is not yet widely available. Gabon is fortunate to have a national health insurance since 2007 that covers 80% of the cost of outpatient and inpatient treatment [14], which allowed us to diagnose Still's disease. In the diagnostic itinerary of our patients, we did not find any consultations with traditional healers, whose treatment is based on scarification and traditional decoctions. SD is a diagnosis of exclusion, requiring the exclusion of infectious, tumor and autoimmune etiologies.

SD causes systemic inflammation, in the early stages of the disease or during relapses and recurrences, resulting from the activation and secretion of pro-inflammatory cytokines, especially interleukin (IL)-1, IL-18 and IL-6 [4, 10, 13]. Nine of our patients (one on acetylsalicylic acid at diagnosis) had hyperferritinemia, as is classically seen in this disease. This very high level of ferritin (as well as IL-8, IL-6, IL-18 and TNF-α) reflects a marked activation of macrophages, which, when activated, synthesize IL-18, which is thought to play a key role in the pathogenesis of the disease [1].

In general, for the same diagnostic criteria [19], type of study (retrospective) and type of population [5, 16], our population remains younger than that observed by other authors in Cameroon, where the mean age of patients is 28 years [16], in Senegal, where it is 43 years [5], and in Tunisia, where it is 35.4 years [3].

Two out of three patients are diagnosed before the age of 40 [15], compared to 9 out of 10 in our series. In 10-15% of cases, MS may be the recurrence of childhood Still's disease. In our patients’ histories, we did not observe any cases of childhood-onset Still's disease that could have led to the relapse of an infantile form. We found 8 out of 10 cases of MS in the dry season, but the seasonal influence on the onset of this disease has never been studied in sub-Saharan Africa. Currently, there is a consensus among scientific societies that adult and juvenile forms of MS are the same disease with the same clinical presentation [8]. A distinction is made between joint-tropic SD and SD with systemic involvement. Clinically, SD is characterized by a triad of a) high fever, b) transient rash, and c) joint involvement with arthralgia or arthritis. Septic fever with chills remains a constant in studies from sub-Saharan Africa [5, 16], as does joint involvement (8 cases in our series), skin involvement (8 cases), and ENT involvement, which was found in 7 of our patients. In its typical form, skin involvement in Still's disease is associated with small macules or maculopapules on Caucasian skin, salmon pink in color, nonpruritic, transient, located on the roots of the limbs, and appearing at the time of febrile flare-ups. This skin condition is more difficult to recognize in black skin and may surprise even experienced specialists (Fig. 2). This may explain the low incidence of skin involvement found in the series of Singwe-Ngandeu *et al.* (16.6%) [16]. In our series, we note, on the one hand, a lower prevalence of liver and lymph node involvement (attributed to SD in view of their regression under corticosteroid therapy) and, on the other hand, an absence of cardiac involvement. However, these disorders are common in SD.

**Figure 2:** Skin lesions of Still's disease on black skin

Biologically, no disturbances are specific to SD, but three are highly suggestive: 1) an inflammatory syndrome, 2) neutrophil hyperleukocytosis and 3) hyperferritinemia with a glycosylated fraction below 20%. In the normal state, the glycosylated form represents 60-80% of the circulating form. In Still's disease, the percentage of the glycosylated fraction is lower than in other inflammatory and auto-inflammatory diseases [7]. This fraction appears lower in the active phase, but also during phases of remission. The combination of hyperferritinemia 5 times higher than normal, coupled with a glycosylated fraction < 20%, corresponds to a specificity of 93% and a sensitivity of 40% for Still's disease [18]. Unfortunately, in our study, due to its high cost compared with patients’ financial possibilities, this biological marker could only be assayed in one patient. It therefore seems to us that the criteria of Yamaguchi *et al.* [19] are more accessible for the diagnosis of MS in sub-Saharan Africa than those of Fautrel *et al.* [9].

In terms of treatment, non-steroidal anti-inflammatory drugs (NSAIDs), which are effective in MS in children, appear to be effective in only 20% of adults with SD in European series [12]. For this reason, we opted for isolated corticosteroid therapy, which could be combined with methotrexate. Corticosteroid therapy was used at a dose of 0.5 to 1 mg/kg/day prednisone in all our patients, with the introduction of methotrexate at a dose of 0.3 mg/kg/day, for the purpose of cortisone sparing, but also for joint involvement, given its proven efficacy in cases of corticoresistance (4/10 patients). Recurrences are less frequent when methotrexate is combined with corticosteroid therapy (8% in the series by Diallo *et al.*) [5]. In the event of therapeutic failure, discussion turns to the introduction of biotherapies. Although innovative and highly effective, these therapies have their limits in sub-Saharan Africa, due to their high cost and the resurgence of tuberculosis that they engender in a continent where the disease is endemic [6].

In all cases, our patients could not be weaned from corticosteroids and methotrexate. Four of our patients developed corticosteroid resistance, requiring the addition of methotrexate, and one died of lymphohistiocytic activation syndrome.

## Conclusions

Data on SD in sub-Saharan Africa remain patchy. Our diagnostic preference is for the criteria of Yamagushi *et al.* [19]. Our study confirms a low incidence of liver and lymph node involvement and an absence of cardiac involvement. This needs to be confirmed by other studies in a larger cohort. Also, the frequency of diagnosis in the dry season (low temperature season) should be studied in a larger cohort.

## Funding

No funding was provided for this work.

## Authors’ contributions

Iba Ba Josaphat: design and editing Mfoumou Annick Flore: editing and proofreading Nseng Nseng Ondo Ingrid: proofreading and data collection

Kanganga Ekomy Arthur: bibliographical research, proofreading

Ledaga Lentombo Léonie Esther, Igala Marielle, and Kombila Ulrich Davy: proofreading Boguikouma Jean Bruno: design, proofreading, approval of final version

## Conflict of interest

The authors declare no conflict of interests
